# Exploring the Long-Term Disability Outcomes in Trauma Patients: Study Protocol

**DOI:** 10.21203/rs.3.rs-4238506/v1

**Published:** 2024-04-10

**Authors:** Natasha Shaukat, Asma Altaf Hussain Merchant, Fazila Sahibjan, Ayesha Abbasi, Zeerak Jarrar, Tanweer Ahmed, Huba Atiq, Uzma Rahim Khan, NadeemUllah Khan, Saima Mushtaq, Shahid Rasul, Adnan Hyder, Junaid Razzak, Adil Haider

**Affiliations:** Aga Khan University; Aga Khan University; Aga Khan University; Aga Khan University; Aga Khan University; Jinnah Postgraduate Medical Center; Aga Khan University; Aga Khan University; Aga Khan University; Jinnah Postgraduate Medical Center; Jinnah Postgraduate Medical Center; George Washington University; Aga Khan University; Aga Khan University

**Keywords:** Patient Reported Outcomes, Digital Trauma Registries, Disabilities, low- and middle- income countries

## Abstract

**Objectives:**

Post-discharge patient-reported outcomes from trauma registries can be used to measure trauma care quality. However, studies reflecting the Asian experience are limited. Therefore, we aim to develop a digital trauma registry to prospectively capture patient-reported outcomes (PROs) at one-, three-, six-, and twelve-months post-injury in Pakistan.

**Methods:**

We will use a cohort study design to develop a digital trauma registry at two tertiary care facilities (Aga Khan University Hospital & Jinnah Postgraduate Medical Center) in Karachi, Pakistan. The registry will include all admitted adult trauma patients (≥18 years). Data collection will be digital using tablets, with mortality, level of disability, and functional status, quality of life being the outcomes. Telephonic interviews will be conducted with the patients and caregivers for follow-up data collection.

**Discussion:**

The high disability burden following accidental trauma imposes a significant burden and cost on individuals and society. Therefore, the trauma registry would fill this gap by capturing post-discharge long-term PROs. It will provide the injured patient’s post-discharge situation, challenges, and future directions for incorporating long-term PROs in low-resource settings. Including long-term measures in routine follow-ups will provide insights into physical, social, and policy barriers and help advance injury care research.

## Introduction

Trauma is a global public health concern. With almost 1 in 10 fatalities, injuries rank among the leading causes of morbidity and mortality worldwide. According to the Global Burden of Disease report from 2019, injuries accounted for 7.6% of global mortality, with traffic fatalities being the leading cause and accounting for 2.9% of disability life-adjusted years (DALYs).^1,2^ In low- and middle-income countries (LMICs), where DALYs are on the rise annually, this injury burden is significantly higher.^2^ There are an estimated 14.3 deaths per 100,000 people in Pakistan from road traffic crashes, with a total of 27,582 (95% CI: 23,343 − 31,920) fatalities.^3,4^ However, the estimate of injury burden is constrained because there isn’t a comprehensive trauma database.^5,6^

One approach for collecting information about injuries is the usage of trauma registries. They are an integral part of advanced trauma systems to promote injury prevention and improve patient outcomes.^7–9^ Evidence supports improved trauma care with well-established registries.^10^ However, most registries are limited by a lack of post-discharge data (survival, functional status, disability), which is essential in understanding the long-term impact of trauma care.^11,12^ Furthermore, most registries focus on mortality as an outcome and few on functional status at discharge.^10,11^

Increased costs associated with setting up a trauma registry and the continuous expenditure of human resources to collect and analyze data have limited LMICs from building their data systems.^11^ Cost-effective measures have been identified, which has led to an increase in the establishment of trauma registries in resource-constrained regions of South Asia, East, and South Africa. However, these systems still lack measures of sustainability and long-term operation.^13^ A scoping review estimates 43 functional registries in LMICs, with only 10 using their data productively.^13^ This depicts a need to focus on assessing long-term disability outcomes in trauma patients to understand its burden and identify solutions required to cater to it.

In Pakistan, a developing country in South Asia, 6% of mortality is caused due to injuries alone, with youth and middle-aged people most susceptible to it.^14^ Trauma registries have been piloted and implemented at various levels in Pakistan, but barriers such as extensive cost and missing data have limited its successful application. Several cross-sectional studies highlight the demographics, causes, and interventions taken for trauma patients in Pakistan,^15,16^ but few efforts have been made to collect long-term outcomes following trauma.^17^ Patient-reported outcomes can bridge this knowledge gap and better inform clinical decision-making and quality improvement efforts.^12^ However, this information is not available in Pakistan.

This study aims to develop a digital trauma registry to prospectively capture patient-reported disability outcomes (PROs) at one-, three-, six- and twelve months post-injury in Pakistan.

## Methods

### Study Design and Setting

We will use a prospective cohort study design. With nearly 212 million residents, Pakistan has the sixth-highest population in the world. Karachi has the eleventh-highest population worldwide. The registry will be established in two major tertiary care hospitals in Karachi, Pakistan. The Aga Khan University Hospital (AKUH) is a private sector hospital, while Jinnah Postgraduate Medical Centre (JPMC) is a public sector hospital.

With an average bed capacity of (AKUH = 760 beds and JPMC = 2000 beds), these teaching hospitals offer tertiary care. Each facility has its own emergency room. Compared to JPMC’s emergency room, which saw over one million patients a year, AKUH saw over 55,000 patients annually. General surgeons, anaesthesiologists, orthopaedic, and neurosurgery surgeons were present in both institutions. Both institutes have developed treatment protocols to address acute emergencies. Over 80% of the necessary supplies and equipment are available in both institutions.

### Eligibility Criteria

All admitted adult trauma patients (≥ 18 years) with one or more traumatic injuries, which is defined as the injury being severe enough to need hospitalization for at least 24 hours will be included. The patients will be included from the wards (orthopaedics/general surgery/thoracic) ICU/HDU. Patients under 18, those unable to communicate verbally without a proxy, and those released from hospital within 24 hours will not be included.

### Sample Size

For the follow-ups we estimated a sample of 1365 individuals, 80% power with an r-square ranging between 0.1 to 0.6, the anticipated odds ratio of 1.75 or more, anticipated probability of having an outcome (functional limitation) of 30% or more at a 0.05 level of significance.^18^

### Planning, needs assessment and stakeholder engagement

We conducted an initial needs assessment to determine the current situation of injury data collection, defined specific data points to be collected, identified challenges/barriers in setting up a trauma registry, and identified solutions to overcome these challenges. Next, we will identify and engage key stakeholders, including hospital leadership, administrators, departments, and clinicians, to get their buy-in on the project.

### The Registry

We will develop a digital trauma registry to assess short, medium, and long-term disability outcomes. We will recruit admitted patients in the hospital using the trauma registry questionnaire. The data will be collected 8–10 hours per day, six days a week, during the morning shift (9 a.m.−5:30 p.m.). In addition, we will follow up with patients telephonically at one, three, six- and twelve months post-discharge.

### The development of trauma registry questionnaire

The study team developed, refined, and finalized the trauma registry questionnaire. We primarily used the Collector Trauma Registry as a guideline to develop the in-hospital registry questionnaire.^19^ We added a few context-specific variables (e.g., ethnicity, occupational classification) related to trauma systems in LMICs. We refined the questionnaire using multiple rounds of discussions with national and international experts (Emergency Medicine physicians, trauma surgeons, and public health experts). The experts gave input on selecting variables, sequencing, language, and outcome measures. Based on the expert’s feedback, outcomes, such as septic complications and duration of stay, were added. We pretested the questionnaire on twenty eligible trauma patients in AKUH. We assessed the individual questions and the overall design of the questionnaire. We identified the necessary changes in sequencing and language and updated the questionnaire. Finally, the modified questionnaire was again administered to a few more patients. The registry is simple, with clear and standardized fields, and uses drop-down menus to minimize errors. The complete set of variables is shown in Table 1.

### Outcome Measures

The outcome measures are discharge outcomes, in-hospital and post-discharge mortality, duration of stay, septic complications, functional status, quality of life (QoL), and psychosocial outcomes (PTSD) (Table 2).

We used the following questionnaires to record patient’s reported outcomes for functional limitations, quality of life, and PTSD at one, three, six-, and twelve-months post discharge follow-ups. The questionnaires were translated into Urdu and later back-translated into English by native Urdu speakers fluent in English and Urdu. We pretested the questionnaire to assess whether the words and terms used in the Urdu version were clear, relevant, and comprehensible.

- Functional Independence Measure (FIM): The Functional Independence Measure (FIM) tool is a fundamental measure of *patient disability*. The 18 items in the FIM instrument comprise six domains, as mentioned in Table 2. A scale of 1 (complete dependence) to 7 (complete independence) is used to rate each item; higher scores signify a higher level of functional independence (summed scores range from 18 to 126). Independent studies have provided evidence of the FIM instrument’s validity and reliability, including self-report through phone interview.^20,21^ An intra-class correlation coefficient of 0.97 indicates good agreement between FIM overall score obtained over the phone and in-person.^21^
- Trauma Quality of Life (TQoL): The revised trauma quality of life (RT-QoL) instrument measures trauma specific long-term quality of life outcomes. This is a five-component, 43-item questionnaire, with the five domains specified in Table 2.^22^
- Primary Care PTSD Screen for DSM: This questionnaire is used to assess post traumatic stress disorder (PTSD). The questionnaire screens with an item which assesses lifetime exposure to traumatic events. If a respondent denies exposure, the PC-PTSD-5 is complete with a score of 0. However, if a respondent indicates that they have had any lifetime exposure to trauma, the respondent is instructed to respond to 5 additional yes/no questions about how that trauma exposure has affected them over the past month.^23^

### Personnel and training

The research team comprises a team lead, a research specialist who coordinates the day-to-day activities, and three medical officers who will collect data. They attended two days of training: one day in class and one-day on-site training. They received training on software use, ICD-10 coding, Abbreviated Injury Scale (AIS), Injury Severity Score (ISS), and Revised Trauma Score (RTS) training. We will also provide frequent refresher training (every 2–3 months) to address data collection challenges and train new data collectors.

### Recruitment

The data collector will be placed in the wards (orthopedics, general surgery, and thoracic) and surgical Intensive Care Unit (ICU)/ High dependency Unit (HDU). With the assistance of nurses, doctors, admission registers, they will identify new admissions and, after obtaining informed consent, and patients meeting the eligibility criteria the subjects will be enrolled in the study. Then, they will interview the patient at the bedside to collect the required basic information. If the patient cannot answer, the caregivers will be interviewed. Next, the data collector will extract detailed information from the medical records (labs, radiology reports, and discharge summary). Finally, at the time of discharge, the patient/caregiver will be re-interviewed. If the patient leaves the hospital before being interviewed, they will be telephonically followed up within a week of discharge to document discharge outcomes.

### Follow-ups

Patients enrolled in the study will be followed across the continuum of recovery. These will comprise twenty to twenty-five-minute telephonic interviews. The interview will have an initial screening, verbal consent, and questions about his/her recovery. The patients will be approached up to ten times, after which he/she will be considered a loss for follow-up.

### Data Entry

We will enter data through digital software (RedCap).^24^ RedCap is a free-of-cost, secure web application for surveys and databases. Online data entry at RedCap is fast, flexible, and easy to use. We will build the project database on RedCap by uploading the data collection tools (trauma registry and follow-up questionnaires). To ensure that data collection tools look and work as we intend, we will create a few test records and enter some data for each tool.

### Quality assurance

The data collectors will use an excel sheet to send daily updates of newly enrolled patients and the number of follow-ups calls through google docs. Additionally, only team members will have access to Google Docs. These forms, however, will report the ID numbers and do not contain any patient identifiers. This information will be stored on a password-protected computer. The research specialist (RS) will close out the cases on Google Documents after verifying them on Redcap. The PI will receive weekly updates and cross check the enrollments on a regular basis. The research specialist will make random visits weekly to oversee data collection, spot and address field problems, and ensure all eligible patients are enrolled in the study by comparing enrolled participants with admission lists and ensure quality checks at field site. The PI will monitor all entered data and check the data for data completeness and accuracy. We will compare the entered data elements with the patient’s medical records for data accuracy. We will calculate the error rate for a subset of records to ensure data quality

### Data analysis

We will conduct a descriptive analysis to summarize the participant profile, clinical characteristics, and outcomes, including the median and interquartile range (IQR), mean and standard deviation, and proportions (95% confidence intervals). Kaplan Meir survival curves and means will be obtained, and the log-rank test will be used to test the hypothesis that survival curves are similar. The coax proportional hazard model (Parametric model) will be used to perform survival analysis. Assumptions of proportionality of hazards will be assessed. We will use univariate logistic regression to assess the association between potential predictor variables and each outcome. Variables with an overall model p-value of < 0.25 will be considered eligible for entering the model-building stage, and a likelihood ratio test will be performed at each step. Multicollinearity among the qualifying variables will be checked using correlation coefficients for quantitative variables and Kramer’s V for categorical variables. Adjusted odds ratios (AOR) and 95% CI will be calculated. The significance level of all statistical tests will be considered at 0.05. Stata for windows version 14 will be used for analysis. For FIM, mean and standard deviation will be reported for one, three, and six months. For TQoL, the mean and standard deviation will be reported for all three sub-components, along with the mean and standard deviation for the overall score.

## Discussion

The study aims to describe the design and methodology of setting up a digital trauma registry to capture long-term disability-related outcomes in Pakistan. It will also provide a comprehensive insight into trauma-related disabilities and how they affect injured patients by looking at their current level of disability and return to work. This will provide an opportunity for recommendations for incorporating PROMs into trauma registries in LMICs to maximize the rehabilitation and reintegration of the injured into society.

### Strengths

This will be among the first studies in Pakistan to collect PROs over a long-time frame following a traumatic injury. This study employs a prospective cohort study design to capture estimates of disability among injured patients using validated instruments at one month, three months, six months, and twelve months following the injury in Pakistan. This study’s large sample size increases the accuracy and generalizability of the findings. In this study, we will calculate the injury severity score and revised trauma score, which are important measures of severe injury. Also, we will assess elements like PTSD that could directly impact outcomes related to disabilities.

### Limitations

The study has some limitations. The study will be carried out in two trauma centres with different volumes, patient flow systems, and resource availability. This might not accurately represent the situation in other local trauma centres in the country. Due to practical reasons, we will not include paediatric trauma patients, missing a substantial proportion of the population contributing to the trauma burden. In addition, we will not fully account for the trauma burden (minor injuries, brought dead, and mortalities in the ED) because we will only include admitted patients. However, since we intend to recruit participants with whom we can follow up to learn more about long-term consequences, collecting the whole trauma burden is not the study’s objective. Some patients will be missed since there will be only eight to ten hours of data collection per day, six days per week. Patients admitted on Sundays or holidays and those who pass away or leave against medical advice at night are only a few examples. There may be differences between missed and admitted patients, and the generalizability may be limited. The extraction of data from medical records may pose certain challenges. Some of the variables in the medical records might be missing or have inconsistent data. Data entry is dependent on the availability of electricity and high-quality internet. There may be challenges related to telephonic follow-ups. Inaccurate phone numbers, inactive phone numbers, female patients’ reluctance to participate, and non-response may pose follow-up barriers.

## Conclusion

The high disability burden following accidental trauma imposes a significant burden and cost on individuals and society. Therefore, the trauma registry would fill this gap by capturing post-discharge long-term PROs. It will provide the injured patient’s post-discharge situation, challenges, and future directions for incorporating long-term PROs in low-resource settings. Including long-term measures in routine follow-ups will provide insights into physical, social, and policy barriers and help advance injury care research.

## Figures and Tables

**Table 1:** Variables included in the in-hospital trauma registry questionnaire

Categories	Variable
Demographic Information	Name, age, medical registration number, gender, ethnicity, contact details, address, education status, occupation, the status of employment prior to the injury, comorbidities, disability status prior to the injury, functional health status prior to the injury
Injury Details	Cause of injury, injury data and time, geographic location of the injury, type of trauma, nature of the injury, International Classification of Diseases (ICD-10) location code (provisional), diagnosis, protective devices, Abbreviated Injury Scale (AIS), Injury Severity Score (ISS), and Trauma Score and Injury Severity Score (TRISS) score
Inter-hospital case management data	Name of prior facility, arrival by, name of ambulance service, treatment if given in ambulance, referral, arrival date at facility, workup done at referring facility, treatment list/medication given in prior facility, procedures done in the prior facility, departure date.
Tracking of patients as he/she moves from ED to OT/HDU/ICU/wards	Location tracking, service tracking, blood transfusions, radiological procedure, surgical procedures, other procedures, and medications.
Outcomes	Discharge outcome, discharge/death date and time, duration of stay, septic complications, disability status at discharge, functional health status at discharge

**Table 2:** In-hospital and Follow-up questionnaires data collection methods and time points (Patient-reported outcomes)

Questionnaire	Content	Data Collection Method	Data Collection Time point
In-hospital Trauma Registry Questionnaire	Demographics, injury details, inter-hospital case management, tracking of patients as he/she moves from emergency (ED) to operation theatre (OT)/High dependency unit (HDU)/ Intensive Care Units (ICU)/wards, Outcomes	Medical Records, Inperson interviews	In-hospital and within one week of discharge
Follow-up questionnaires at one, three, six-, and twelve-months post discharge			
Functional Independence Measure (FIM)	Self-care, sphincter control, transfer, locomotion, communication, and social cognition	Telephonic Interview with patient/proxy	Within one week of discharge, one, three, six-, and twelve-months post discharge
Trauma Quality of Life (TQoL)	Emotional well-being, recovery and resilience, physical well-being, functional engagement, and peri-traumatic experience	Telephonic Interview with patient/proxy	One, three, six-, and twelve-months post discharge
Primary Care PTSD Screen for DSM	PTSD	Telephonic Interview with patient/proxy	One, three, six-, and twelve-months post discharge

## Abbreviations

PROs: Patient-Reported Outcomes
ED: Emergency Department
AIS: Abbreviated Injury Scale
ISS: Injury Severity Score
TRISS: Trauma Score and Injury Severity Score
QoL: Quality of Life
PTSD: Post-Traumatic Stress Disorder
